# Applying Fourier Transform Mid Infrared Spectroscopy to Detect the Adulteration of *Salmo salar* with *Oncorhynchus mykiss*

**DOI:** 10.3390/foods7040055

**Published:** 2018-04-05

**Authors:** Nuno Sousa, Maria João Moreira, Cristina Saraiva, José M. M. M. de Almeida

**Affiliations:** 1 School of Agrarian and Veterinary Sciences, CECAV, University of Trás-os-Montes e Alto Douro, Quinta de Prados, B. Laboratorial, B1.03, 5001-801 Vila Real, Portugal; nunofilipe_sousa@hotmail.com (N.S.); mjoaom24@hotmail.com (M.J.M.); crisarai@utad.pt (C.S.); 2 Physics Department, School of Sciences and Technology, University of Trás-os-Montes e Alto Douro, Quinta do Prados, 5001-801 Vila Real, Portugal; 3 CAP/INESC TEC—Technology and Science and FCUP-Faculty of Sciences, University of Porto, 4169-007 Porto, Portugal

**Keywords:** food fraud, food authentication, *Salmo salar* adulteration, FTIR spectroscopy, chemometrics methods

## Abstract

The aim of this study was to evaluate the potential of Fourier transform infrared (FTIR) spectroscopy coupled with chemometric methods to detect fish adulteration. Muscles of Atlantic salmon (*Salmo salar*) (SS) and Salmon trout (*Onconrhynchus mykiss*) (OM) muscles were mixed in different percentages and transformed into mini-burgers. These were stored at 3 °C, then examined at 0, 72, 160, and 240 h for deteriorative microorganisms. Mini-burgers was submitted to Soxhlet extraction, following which lipid extracts were analyzed by FTIR. The principal component analysis (PCA) described the studied adulteration using four principal components with an explained variance of 95.60%. PCA showed that the absorbance in the spectral region from 721, 1097, 1370, 1464, 1655, 2805, to 2935, 3009 cm^−1^ may be attributed to biochemical fingerprints related to differences between SS and OM. The partial least squares regression (PLS-R) predicted the presence/absence of adulteration in fish samples of an external set with high accuracy. The proposed methods have the advantage of allowing quick measurements, despite the storage time of the adulterated fish. FTIR combined with chemometrics showed that a methodology to identify the adulteration of SS with OM can be established, even when stored for different periods of time.

## 1. Introduction

Atlantic salmon (SS) is economically important in the daily life of consumers, since it is a good source of polyunsaturated fatty acids, specifically two important omega-3 fatty acids: eicosapentaenoic acid (EPA) and docosahexaenoic acid (DHA) [1,2]. They are composed of 5–10% red muscle and 90–95% white muscle [3]. The red/orange color is due to the presence of carotenoid pigment, named astaxanthin, which has antioxidant activity, leading to a high oxidative stability [4].

Salmon trout (*Onconrhynchus mykiss*) (OM) and SS are visually similar, namely in muscle color, as well as rich in EPA and DHA. They are the major species of European aquaculture because of the pressure on the wild fish population. Consequently, access to these species has become limited [5]. The pigmentation of OM is caused by the keto-carotenoids astaxanthin and canthaxanthin [6].

In the last decade, the issue of food safety has acquired increased importance, due to rapid changes in the agro-food system. Fraud is a major concern for the food industry. Fraud is defined as the intentional act of substituting, adding, adulterating, tampering, or misrepresentation of ingredients, and/or packaging [7]. This not only decreases the quality of products, but also misleads consumers and may involve associated health risks [8,9]. There are different types of food adulteration, namely unauthorized partial or total substitution of commercial valuable species with cheaper products [10], frozen-thawed product sold as fresh [11,12], classification fraud of species or origin [13], and the presence of genetically modified organisms.

In the past, a variety of standard analytical methods were applied to detect the adulteration of proteins, such electrophoresis (polyacrylamide gel electrophoresis), immunological analysis (immuno-diffusion techniques, immuno-electrophoresis, and linked immune-adsorption assays), and chromatographic and DNA-based procedures (polymer-chain reaction) [14]. However, these methods require skilled technicians and a relatively long time for sample preparation and analysis [7].

Presently, innovative and non-destructive spectroscopy techniques are being developed. These techniques require small samples and no complex preparation is necessary, thus allowing simple, fast, and accurate measurements [15,16]. Emerging non-destructive mapping technologies for authentication and traceability include visible/near infrared, mid infrared, fluorescence spectroscopy [17], and Raman spectroscopy (RS), sometimes coupled with the Fourier transform infrared (FTIR) technique.

FTIR spectroscopy has substantial potential as a quantitative method in the food industry. When used together with an attenuated total reflectance (ATR) module and chemometric, FTIR offers methodologies capable of qualitatively and quantitatively discriminating foodstuff based on the spectral characteristics of the food matrix [17,18].

Chemometrics use mathematical and statistical techniques to select the best experimental procedure and treatment of chemical analysis data [19]. There are several chemometrics methods applied to spectroscopy, namely partial component analysis (PCA), discriminant analysis, principal least squares discriminant analysis, and partial least squares regression (PLS-R), among others [17].

There are few studies that quantify fish adulteration using FTIR spectroscopy coupled with chemometrics. This study explores the potential of FTIR as a rapid and accurate method to detect and predict the adulteration of SS with OM, regardless of their storage period.

## 2. Material and Methods

### 2.1. Sampling

SS and OM fish were eviscerated, skin removal was carried out, and muscle was crushed separately in a mincer under sterilized conditions. Mini-burgers of SS adulterated with OM, from 0 to 100% *w*/*w* in steps of 10% *w*/*w*, were produced. For each sampling point, four mini-burgers were produced, two for fat extraction and FTIR and two for microbiological analysis.

The mini-burgers, weighing approximately 15 g, were prepared by mixing the fish and later packed in air overwrapped with polyethylene film. Following packaging, samples were stored at 3 °C and examined for microbiological parameters at intervals of 0, 72, 160, and 240 h.

The microorganisms analyzed were total mesophilic (TVC) and psychrotrophic (TP). In addition, after each predefined storage period, the samples were submitted to Soxhlet extraction and the extracted lipids were analyzed by FTIR.

The experiment was repeated four times, each batch having 176 samples, totaling 704 mini-burgers: 352 for FTIR measurements and 352 for microbiological determinations.

### 2.2. Microbial Analysis

Samples were homogenized with tryptone salt broth (tryptone 0.1% and NaCl 0.85%) in a stomacher for 90 s. Serial decimal dilutions were prepared in the same solution for microbiological determinations. TVC [20] and TP [21] populations were obtained after incubation on plate count agar (PCA) (Oxoid CM0325, London, UK) at 30 °C for 3 days and 7 °C for 10 days, according to ISO4833 of 2003 and NP2007 of 1987, respectively.

### 2.3. Determination of Moisture Content

The measurement of the moisture content consisted in drying the samples in an oven at 100 °C. The weight of the samples was controlled at 60-min intervals using an analytical balance with a resolution of 0.001 g. The process stopped when the mass of the last two weightings, separated by 60 min, did not differ by more than 0.1%. The samples were then stored in a desiccator with silica.

### 2.4. Determination of Free Fat Content/Soxhlet Extraction

Fat extraction was carried out by n-hexane in the dehydrated samples. The dried sample and traces of the sample on the Petri dish were removed using cotton wool moistened with n-hexane and later placed in an extraction thimble. Then, the extraction thimble was positioned in the extraction tubes together with n-hexane, and a flask was adapted to the extractor apparatus.

The extraction process lasted 8 h, after which the flask was placed in a water bath at 90 °C to remove n-hexane, leaving only the fat. After this process, the flask was placed in the oven for 1 h at 103 °C to remove n-hexane residues. These procedures (drying and weighing) were repeated until the results of both successive weightings, separated by 1 h, did not differ by more than 0.1% [22].

### 2.5. Fourier Transform Infrared Measurement

The infrared absorption spectra were collected in a FTIR spectrometer (Shimadzu, Tokyo, Japan) equipped with an ATR module (Golden Gate, Specac Ltd., Orpington, UK), a DLaTGS detector, and a KBr beam-splitter.

Samples of fish fat were placed on top of the ATR crystal, whose temperature was set to ~35 °C. The collection time for each sample spectrum was approximately 2 min. The spectrum was recorded in the region between 4000 and 500 cm^−1^ with a resolution of 4 cm^−1^ and 32 scans. In the ATR module, the infrared radiation underwent total internal reflection when the incident angle at the interface between the sample and the crystal was higher than the critical angle, which is a function of the refractive indices of the two surfaces, allowing the penetration of radiation into the sample [18]. The ATR base was carefully cleaned in situ by scrubbing with pure ethanol (Sigma Aldrich, Taufkirchen, Germany) before measuring the next sample. For each sample, two spectra were collected and the average was calculated.

### 2.6. Mathematical Treatment

#### 2.6.1. Principal Component Analysis

Spectral data collected between 500 and 4000 cm^−1^ were divided into two ranges, from 650 to 1850 and from 2800 to 3050 cm^−1^. Spectral dataset was initially submitted to smoothing based on the Savitzky-Golay algorithm. Following this, the data were mean-centered and standardized (SNV) [23]. 

For a preliminary exploration, the spectral dataset was handled by PCA, which allowed determining its main features as well as highlighting relations among the original variables (absorbance at different wavenumbers). The PCA projects the large number of potentially correlated original variables in a representation space of smaller dimensions and calculates new variables, called principal components (PC), that are linear combinations of the starting absorbances and thus reduce the size of the dataset [24].

#### 2.6.2. Partial Least Squares Regression

For quantitative analysis, the measured factors, contributing to the variance of the dataset, were regressed using PLS-R onto the referred variables [25,26]. This multivariate calibration technique, sometimes called factor analysis, transformed the original variables (FTIR spectra absorbencies) into new ones (known as latent variables), which are linear combinations of the original variables [27]. The method relied on two phases: the so-called calibration and cross-validation steps. Calibration consists in building a mathematical model to establish a correlation between the matrix of FTIR spectra (predictor variables, *X*) and the concentration of analytes of interest (response variables, *Y*) which use a set of observations usually named the calibration set. Cross-validation is performed by using the calibration model to calculate the concentration of samples not used to set up the model [28].

The relative performance of the established model was accessed by the root mean square error of calibration (RMSEC), root mean square error of cross-validation (RMSECV), and multiple coefficient of determination or regression coefficient (*R*^2^) [29]. The selected model was then used to determine the concentration of samples in an independent prediction set. The predictive ability of the model was evaluated from the root mean square of prediction (RMSEP). The lower the RMSEP value, the higher the degree of accuracy of the prediction result provided by the calibration model [30].

PCA, DA, and PLS-R calculations were performed using the Excel-based XLSTAT V2006.06 package (Addinsoft, Inc., New York, NY, USA) and statistical software Unscrambler V9.6 package (Camo, Oslo, Norway).

## 3. Results and Discussion

### 3.1. Microbial Analysis

Table 1 shows the evolution of TVC and of TP with storage time for pure SS and pure OM. The TVC and TP counts increase with storage time, as expected, following and exponential growth. Both species have very similar counts at time 0, but after 240 h pure OM showed a more pronounced development of both TVC and TP.

### 3.2. Determination of Moisture Content

Prior to fat extraction, the samples were dehydrated. They were weighed hourly during the drying process to determine the evolution of water loss. The SS samples have a slightly higher relative humidity value compared to OM samples, 66.44 and 64.76%, respectively, at time 0. 

The SS samples retained more water, so its loss was more pronounced during first few minutes of drying. However, the longer storage time, in this case at 3 °C, the greater the loss of moisture upon the first 60 min of drying. This is due to the interaction of lipid oxidation with proteins, which causes the loss of water retention capacity [31]. Therefore, the loss of water in the first 60 min is higher in samples with longer storage times compared to that in the most deteriorated ones, due to the increased water availability.

### 3.3. Determination of Free Fat Content/Soxhlet Extraction

To determine the fat content, the Soxhlet method was used. Table 2 shows that OM samples had a higher percentage of fat than SS samples. Thus, OM is more susceptible to lipid oxidation, which may lead to a more pronounced deterioration than SS.

### 3.4. Fourier Transform Infrared Measured Spectra

#### 3.4.1. Preliminary Analysis of the Spectral Dataset

**Figure 1:**
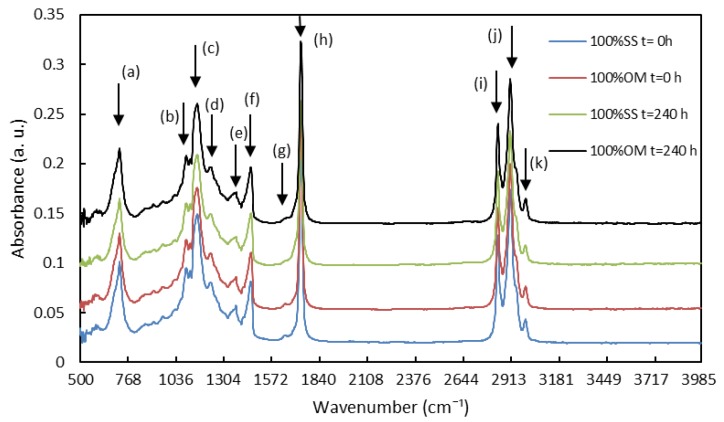
Fourier transform infrared spectroscopy (FTIR) spectra of fat extracted from fresh samples of *Onconrhynchus mykiss* (OM) and *Salmo salar* (SS) and stored at 3 °C for 240 h (*y*-axis).

Figure 1 shows the absorption spectra of the fat extracted from the samples in the medium infrared region between 500 and 4000 cm^−1^ of the pure samples at time 0 and 240 h. Several peaks, which correspond to different functional groups, can be observed in Figure 1. Meanwhile, Table 3 outlines the principal peaks and presents their origins [32,33,34].

The appropriateness to perform PCA was confirmed by Bartlett’s sphericity test (*p* < 0.0001). The number of components retained in the final solution was based on the Kaiser-Meyer-Olkin (KMO) criterion for the analysis of eigenvalues (>1) and the proportion of variance retained (>70%), usually seen as the minimum needed to make the model suitable for explaining the original data. The FTIR spectroscopic data corresponding to the various adulteration levels of SS with OM were subjected to PCA. The KMO sample adequacy measurement was 0.859, which means that the suitability of the sample was good.

**Figure 2:**
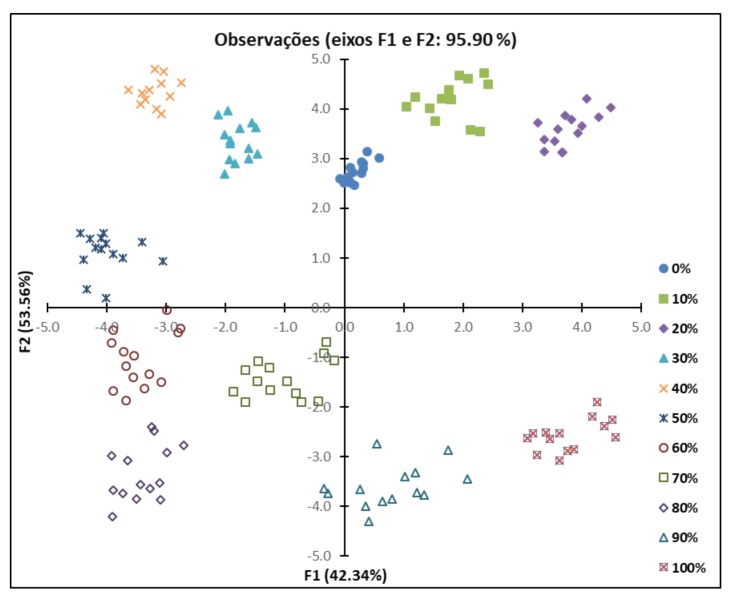
Observations diagram obtained by principal component analysis (PCA) using the Fourier transform infrared spectroscopy (FTIR) spectral data for the 11 formulations of *Onconrhynchus mykiss* (OM) and *Salmo salar* (SS).

It was concluded that 36 PCs describe the variance of the dataset represented by the original variables. It was observed that 95.90% of the variance was explained by only two main components, F1 and F2 principal components describing 42.34% and 53.56% of the variation, respectively. PCA was used to verify the possibility of using FTIR to distinguish SS samples with different adulteration levels of OM. Figure 2 shows the graph of observations for components F1 and F2. It can be observed that the samples with the same percentages of adulteration are grouped into clusters, regardless of storage time.

#### 3.4.2. Partial Least Squares Regression Models for Prediction Based on Spectral Dataset

PLS-R calibration was performed to determine the feasibility of establishing a relationship between the predictive variables (*x*, absorbances) and the percentage of adulteration (*y*, response variables).

PLS-R was performed using the frequency regions of two ranges, from 650 to 1850 cm^−1^ and from 2800 to 3050 cm^−1^. It was then performed on the same frequency regions used for PCA. The quality of the fitting was scrutinized by RMSEC, multiple *R*^2^, and RMSECV.

To validate the developed PLS-R models, leave-one-out cross-validation (LOOCV) was the method applied to a training set of 341 samples to evaluate the adequacy of the PLS-R technique. One sample at a time was randomly excluded. Then, the properties of the removed sample were predicted with a model constructed with the remaining samples (the training set). This procedure was repeated until each sample was excluded once [35]. The ability of models to predict the properties of a set of 11 samples not used to construct the model (the external set) was inspected by evaluating the RMSEP.

**Figure 3:**
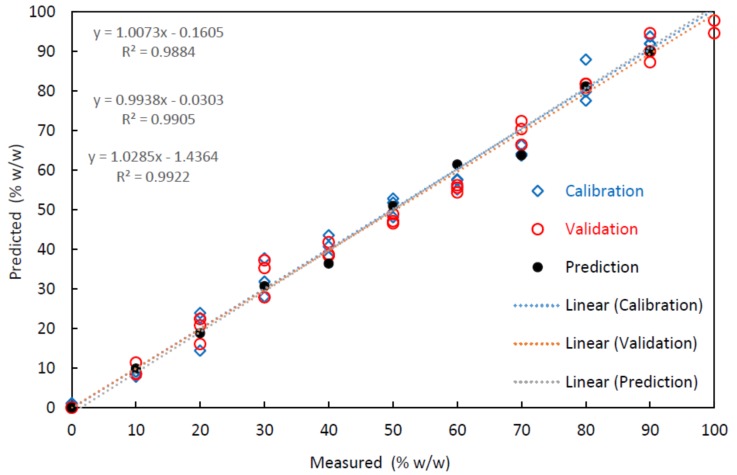
Illustration of the quality of prediction models obtained by Fourier transform infrared spectroscopy (FTIR) for the observed and estimated values for the different mixtures of *Salmo salar* (SS) and *Onconrhynchus mykiss* (OM).

Table 4 presents the quantitative performance of multivariate calibrations determined in this study, in terms of multiple *R*^2^, RMSEC, RMSECV, and RMSEP. The high value of *R*^2^ and the low values of RMSE indicate the good accuracy and precision of the PLS-R models [36]. The values of *R*^2^ and RMSE were 0.988 and 5.6 *w*/*w*, respectively, for calibration. When data were subjected to cross-validation, the RMSE increased to 6.7 *w*/*w*. However, in the calculation of the adulteration level of the external set of 11 samples, the value of the RMSEP increased to 8.7 *w*/*w*. Figure 3 illustrates the accuracy and performance of the models that correlate the measured and estimated adulteration values from the FTIR dataset.

## 4. Conclusions

The use of FTIR coupled with chemometric methods allowed us to accurately estimate the percentage of adulteration of SS with OM. The process of classification using PLS-R allowed the discrimination of samples at 10 levels of adulteration, in steps of 10%, and was successful carried out using fresh samples as well as samples stored for different periods of time, and at diverse stages of the deterioration process.

## Figures and Tables

**Table 1:** Total mesophilic (TVC) and total psychrotrophic (TP) microorganism counts (mean and standard deviation) in *Salmo salar* (SS) and *Onconrhynchus mykiss* (OM) samples, according to storage period.

Micro-Organisms	Fish Species	Time (h)			
		0	72	168	240
TVC	SS	3.44 ± 0.46	4.67 ± 0.10	6.82 ± 0.23	7.75 ± 0.22
	OM	3.89 ± 0.61	6.26 ± 1.12	7.98 ± 0.25	8.81 ± 0.21
TP	SS	3.19 ± 0.52	4.61 ± 0.03	6.16 ± 0.06	7.47 ± 0.25
	OM	3.89 ± 0.61	5.39 ± 0.31	8.00 ± 0.25	8.86 ± 0.21

**Table 2:** Fat content of *Salmo salar* (SS) and *Onconrhynchus mykiss* (OM) samples in (% ***w***/***w***).

Mixture (% *w*/*w* of OM/SS)	Fat Content (% *w*/*w*)
0	11.75 ± 0.78
10	11.62 ± 0.71
20	11.71 ± 1.11
30	12.6 ± 1.34
40	12.75 ± 0.81
50	12.62 ± 0.67
60	13.31 ± 0.71
70	13.15 ± 1.19
80	13.78 ± 0.66
90	14.21 ± 0.57
100	13.65 ± 1.35

Values of mixture are % *w*/*w* of OM in SS.

**Table 3:** Assignment of functional groups present in *Salmo salar* and *Onconrhynchus mykiss* fat responsible for infrared absorption.

Assignment	Wavenumber (cm^−1^)	Functional Group Responsible for IR Absorption
(a)	721	*cis*-disubstituted olefins (–CH_2_–, –HC=CH– (cis))
(b)	1097	ester of the –C–O group
(c)		–C–O, CH_2_ groups and are correlated with saturated acyl groups
(d)		–C–O, CH_2_ groups and are correlated with saturated acyl groups
(e)	1370	CH_3_ group
(f)	1464	CH_2_ and CH_3_
(g)	1655	unsaturated acyl group (–C=C–)
(h)		C=O group of triglycerides
(i)	2850 to 2925	symmetrical and asymmetric methylene (CH_2_)
(j)	2850 to 2925	symmetrical and asymmetric methylene (CH_2_)
(k)	3009	*cis* olefinic CH double bonds (=C–H)

**Table 4:** Quality parameters of the multivariate model for the quantification of adulteration of mixtures of *Salmo salar* (SS) and *Onconrhynchus mykiss* (OM). RMSE: Root Mean Square Error.

Number of Factors	*R* ^2^			RMSE (% *w*/*w* of OM/SS)		
	Calibration	Validation	Prediction	Calibration	Validation	Prediction
4	0.988	0.991	0.992	5.6	6.7	8.7

## References

[1] Castejón D. (2016). NMR-detection of methylamine compounds in Atlantic salmon (*Salmo salar*) subjected to E-beam irradiation. Food Control.

[2] Haq M. (2017). Modifications of Atlantic salmon by-product oil for obtaining different ω-3 polyunsaturated fatty acids concentrates: An approach to comparative analysis. J. Food Drug Anal..

[3] Cai L. (2014). Effects of different freezing treatments on physicochemical responses and microbial characteristics of Japanese sea bass (*Lateolabrax japonicas*) fillets during refrigerated storage. LWT Food Sci. Technol..

[4] Fidalgo L.G. (2018). Microbial and physicochemical evolution during hyperbaric storage at room temperature of fresh Atlantic salmon (*Salmo salar*). Innov. Food Sci. Emerg. Technol..

[5] Lundebye A.-K. (2017). Lower levels of Persistent Organic Pollutants, metals and the marine omega 3-fatty acid DHA in farmed compared to wild Atlantic salmon (*Salmo salar*). Environ. Res..

[6] Choubert G., Baccaunaud M. (2006). Colour changes of fillets of rainbow trout (*Oncorhynchus mykiss* W.) fed astaxanthin or canthaxanthin during storage under controlled or modified atmosphere. LWT Food Sci. Technol..

[7] Rady A., Adedeji A. (2018). Assessing different processed meats for adulterants using visible-near-infrared spectroscopy. Meat Sci..

[8] Nunes K.M. (2016). Detection and characterisation of frauds in bovine meat in natura by non-meat ingredient additions using data fusion of chemical parameters and ATR-FTIR spectroscopy. Food Chem..

[9] Spink J., Moyer D.C., Whelan P. (2016). The role of the public private partnership in Food Fraud prevention—Includes implementing the strategy. Curr. Opin. Food Sci..

[10] Boyacı İ.H. (2014). A novel method for discrimination of beef and horsemeat using Raman spectroscopy. Food Chem..

[11] Cheng J.-H. (2015). Integration of classifiers analysis and hyperspectral imaging for rapid discrimination of fresh from cold-stored and frozen-thawed fish fillets. J. Food Eng..

[12] Ottavian M. (2013). Foodstuff authentication from spectral data: Toward a species-independent discrimination between fresh and frozen–thawed fish samples. J. Food Eng..

[13] Standal I.B., Axelson D.E., Aursand M. (2010). 13C NMR as a tool for authentication of different gadoid fish species with emphasis on phospholipid profiles. Food Chem..

[14] Al-Kahtani H.A., Ismail E.A., Asif Ahmed M. (2017). Pork detection in binary meat mixtures and some commercial food products using conventional and real-time PCR techniques. Food Chem..

[15] Kamruzzaman M., Makino Y., Oshita S. (2015). Non-invasive analytical technology for the detection of contamination, adulteration, and authenticity of meat, poultry, and fish: A review. Anal. Chim. Acta.

[16] Alamprese C. (2013). Detection of minced beef adulteration with turkey meat by UV–vis, NIR and MIR spectroscopy. LWT Food Sci. Technol..

[17] Lohumi S. (2015). A review of vibrational spectroscopic techniques for the detection of food authenticity and adulteration. Trends Food Sci. Technol..

[18] Beasley M.M. (2014). Comparison of transmission FTIR, ATR, and DRIFT spectra: Implications for assessment of bone bioapatite diagenesis. J. Archaeol. Sci..

[19] Roggo Y. (2007). A review of near infrared spectroscopy and chemometrics in pharmaceutical technologies. J. Pharm. Biomed. Anal..

[20] International Organization for Standardization (2003). Microbiology of Food and Animal Feeding Stuffs—Horizontal Method for the Enumeration of Microorganisms—Colony-Count Technique at 30 °C.

[21] (1988). NP 2307:1987 = Microbiologie alimentaire: Directives Générales Pour le Dénombrement de Micro-Organismes Psychrotrophes/Instituto Português da Qualidade; elab. CT 61.-Lisboa: Instituto Português da Qualidade. http://biblioteca.esa.ipcb.pt/NormasPortuguesas.pdf.

[22] International Organization for Standardization (1996). Meat and Meat Products—Determination of Free Fat Content.

[23] Savitzky A., Golay M.J. (1964). Smoothing and differentiation of data by simplified least squares procedures. Anal. Chem..

[24] Abdi H., Williams L.J. (2010). Principal component analysis. Wiley Interdiscip. Rev. Comput. Statist..

[25] Liang Y.-Z., Kvalheim O.M. (1996). Robust methods for multivariate analysis—A tutorial review. Chemom. Intell. Lab. Syst..

[26] Wentzell P.D., Montoto L.V. (2003). Comparison of principal components regression and partial least squares regression through generic simulations of complex mixtures. Chemom. Intel. Lab. Syst..

[27] Miller J.N., Miller J.C. (2005). Statistics and Chemometrics for Analytical Chemistry.

[28] De Luca M. (2009). Multivariate calibration techniques applied to derivative spectroscopy data for the analysis of pharmaceutical mixtures. Chemom. Intell. Lab. Syst..

[29] Divya O., Mishra A.K. (2007). Combining synchronous fluorescence spectroscopy with multivariate methods for the analysis of petrol–kerosene mixtures. Talanta.

[30] Corgozinho C.N., Pasa V.M., Barbeira P.J. (2008). Determination of residual oil in diesel oil by spectrofluorimetric and chemometric analysis. Talanta.

[31] Tironi V.A., Tomás M.C., Añón M.C. (2010). Quality loss during the frozen storage of sea salmon (*Pseudopercis semifasciata*). Effect of rosemary (*Rosmarinus officinalis* L.) extract. LWT Food Sci. Technol..

[32] Rohman A., Erwanto Y., Man Y.B.C. (2011). Analysis of pork adulteration in beef meatball using Fourier transform infrared (FTIR) spectroscopy. Meat Sci..

[33] Carton I., Goicoechea E.N., Uriarte P.S. (2008). Characterization of cod liver oil by spectroscopic techniques. New approaches for the determination of compositional parameters, acyl groups, and cholesterol from 1H nuclear magnetic resonance and Fourier transform infrared spectral data. J. Agric. Food Chem..

[34] Guillén M.D., Ruiz A., Cabo N. (2004). Study of the oxidative degradation of farmed salmon lipids by means of Fourier transform infrared spectroscopy. Influence of salting. J. Sci. Food Agric..

[35] Picard R.R., Cook R.D. (1984). Cross-validation of regression models. J. Am. Stat. Assoc..

[36] Huishan L. (2005). Application Fourier transform near infrared spectrometer in rapid estimation of soluble solids content of intact citrus fruits. 2005 ASAE Annual Meeting.

